# African Swine Fever in Saxony—Disease Dynamics

**DOI:** 10.3390/v16121894

**Published:** 2024-12-09

**Authors:** Katja Schulz, Sandra Blome, Michael Richter, Tessa Carrau, Christoph Staubach, Carola Sauter-Louis

**Affiliations:** 1 Friedrich-Loeffler-Institut Institute of Epidemiology, Südufer 10, 17493 Greifswald-Insel Riems, Germany; christoph.staubach@fli.de (C.S.); carola.sauter-louis@fli.de (C.S.-L.); 2 Friedrich-Loeffler-InstitutInstitute of Diagnostic Virology, Südufer 10, 17493 Greifswald-Insel Riems, Germany; tessa.carrau@gmail.com (T.C.); sandra.blome@fli.de (S.B.); 3 State Administration of Saxony, Department 25, Veterinary Affairs and Food Control, 01099 Dresden, Germany; michael.richter@lds.sachsen.de

**Keywords:** surveillance, epidemiological course, prevalence, wild boar

## Abstract

African swine fever (ASF) emerged in Germany in 2020. A few weeks after the initial occurrence, infected wild boar were detected in Saxony. In this study, data from wild boar surveillance in Saxony were analyzed. The analysis focused on the eastern districts of the state, where the disease likely spread through infected wild boar. Additionally, data from Meissen, located approximately 65 km further west, were examined. In Meissen, the disease emerged one year later, and a human-mediated introduction was suspected. To evaluate the progression of ASF in the two study areas over time, data from active and passive surveillance were analyzed both descriptively and using a Bayesian space–time model. Prevalence estimates were calculated for wild boar testing positive for the ASF virus and for ASF-specific antibodies. Higher prevalence estimates were observed in the eastern districts, indicating a stronger viral load, consistent with patterns seen in other European regions. Over time, seroprevalence increased, suggesting an accumulation of surviving wild boar. The findings provide important insights into the epidemiology of ASF in wild boar over time. They complement the existing knowledge and support targeted ASF control measures. This is particularly significant, as ASF continues to spread across Europe rather than being successfully eradicated.

## 1. Introduction

African swine fever virus (ASFV) genotype II has now been present in EU countries for more than 10 years [1]. In most affected countries, infected wild boar play the leading role in the epidemiology and the spread of the disease [2,3]. However, the situation is notably different in countries where domestic pigs are predominantly kept in backyards and raised for private consumption due to cultural practices [4]. The situations in the different countries also vary depending on the epidemiological scenario [5]. Both, the Baltic countries and Germany face a persistent risk of new ASF incursions from neighboring countries also affected by the disease, such as Ukraine, the Russian Federation, and Poland. In contrast, there are repeated cases of ASF in wild boars several hundred kilometers away from the rest of the epidemic (e.g., in Belgium in 2018 [6] or in the Czech Republic in 2017 [7]). Usually, these cases result from human activities. Depending on the epidemiological scenario, also the epidemiological course of the disease, the applied control measures and their success seem to differ [5].

In September 2020, the first ASF cases were detected in Brandenburg, Germany [8]. Just a few weeks later, the district of Görlitz in the federal state of Saxony also reported wild boar infected with ASF. It is believed that these outbreaks were caused by infected wild boar crossing over from neighboring Poland.

However, in 2021, infected wild boar were also detected in the district Meissen, approx. 65 km away from the epidemiological front in the East. Considering the estimated speed of disease spread, it is highly likely that the virus was introduced into this new area by human activity rather than through migrating wild boar [9]. Thanks to the immense effort of all involved stakeholders in the applied control measures, ASF has not moved westwards. As of the time of writing, Saxony managed to prevent a transmission into domestic pig holdings.

In an infected wild boar, the ASFV genome is usually detectable approx. 4 days after the infection, while the immune response in the form of antibodies is detectable after 7–10 days [10,11]. As long as the viral genome is detected, the occurrence of antibodies has no predictive value for the final disease outcome [10]. Several studies demonstrated that the course of ASF within the wild boar population is comparable across affected countries [1,12,13,14]. At the beginning of the epidemic, virus prevalence increases, especially in wild boar carcasses. Later, seroprevalence rises, primarily due to the cumulation of surviving animals. Over time, the prevalence of the virus declines, primarily due to a significant reduction in the wild boar population, which decreases the number of susceptible hosts [15]. As the population eventually recovers, the number of susceptible animals increases once more, often leading to a resurgence of the virus [1,12,13,14].

Monitoring these parameters over time enhances the scientific knowledge about ASFV, its behavior within a population and potential adaptions, attenuations, or other changes. Furthermore, such studies allow the evaluation of the epidemiological course of specific situations, facilitating the design of tailored surveillance and control measures. In the present study, we looked at the spatial and temporal courses of both virus- and seroprevalence in wild boar in Saxony. Thus, we aimed to investigate the epidemiological course of ASF and to evaluate the importance of these parameters in assessing the disease situation in an affected area.

## 2. Materials and Methods

### 2.1. Study Area and Period

Two areas were investigated: area East and area West. In area East, ASF surveillance data were used from the eastern districts Bautzen and Görlitz. However, only data from wild boar located in areas that were defined as restricted zone II on 19 May 2023 were used (Commission Implementing Regulation (EU) 2023/1080 of 2 June 2023 amending Annexes I and II to Implementing Regulation (EU) 2023/594 laying down special control measures for African swine fever and repealing Implementing Decision (EU) 2023/985). Restricted zone II is one of the designated containment areas used to control and prevent the spread of ASF in affected regions. This zone is established around areas where ASF cases have been confirmed in wild boar populations but not yet in domestic pigs. The main objective of restricted zone II is to prevent the disease from spreading to pig farms by limiting the movement and activities in the zone. Thus, the key characteristics of restricted zone II include movement restrictions, increased surveillance and biosecurity measures, wild boar population management, and increased public awareness campaigns.

Municipalities that were only partly located in the restriction zone were included completely. Data were used from 1 October 2020–17 May 2023.

In area West, data were used from the district Meissen using the same approach as with area East. Data from the city of Dresden were also included in this study. The study period was from 1 October 2021–17 May 2023.

### 2.2. Data

Surveillance data were used with the approval of the competent authority and originated from the CSF/ASF wild boar surveillance database of the European Union (https://surv-wildboar.eu) (accessed on 1 July 2023). The data compilation of each data record are described in detail by Richter et al. [9]. For our analyses, we used the location of the sampled wild boar based on x and y coordinates and the corresponding municipalities, the laboratory test result (PCR result for virus genome detection [virology] and ELISA result for antibody detection [serology]), the date and the origin of the sample (from hunted wild boar, wild boar found dead, shot sick, or died from a road traffic accident (RTA)). Additionally, we used the age and sex of the sampled wild boar for analyses.

### 2.3. Data Analyses

The analyses were conducted separately for each study area. Surveillance data were analyzed descriptively using the software package R, version 4.1.2 [16]. Sample sizes for each year and per month were calculated based on samples originating from wild boars found dead, shot sick, died in an RTA, and from hunted wild boars. The results were visualized using the R package “ggplot2” [17].

For the following analyses, data from hunted wild boar and animals that died in an RTA were summarized and analyzed together as one variable, and data from wild boar found dead and shot sick were summarized in a second variable. For every municipality in the affected areas, the median virus prevalence of hunted wild boar and wild boar found dead as well as the seroprevalence (tested positive for ASF-specific antibodies but negative for the ASFV genome) of all hunted wild boar over the study period were calculated. The data were mapped using ArcGIS ArcMap10.8.1 (ESRI, Redlands, CA, USA). Furthermore, monthly ASF prevalence estimates were computed for each study month. In addition to the ASFV- and seroprevalence estimates, prevalence estimates were calculated for wild boar (hunted and found dead) that tested ASFV-positive and simultaneously seropositive. For all prevalence estimates, the 95% confidence interval was calculated [18].

Temporal and spatial effects were calculated with a Bayesian space–time model using BayesX 2.0.1 (http://www.uni-goettingen.de/de/bayesx/550513.html, accessed on 27 June 2024). The model is fully described in Staubach et al. [19] and Staubach et al. [20]. Due to the low number of data in study area West, the model was only applied to area East. The temporal and the spatial course were calculated on a monthly basis and at municipality level, respectively. The application of the model was described previously by Nurmoja et al. [21]. In the model, prevalence constituted the dependent variable, while time, space, and season were included as random factors [12]. The model output represents the temporal or spatial effect on the logit of prevalence, providing insights into how prevalence rates vary over time or across different spatial regions. By modeling the logit-transformed prevalence, we were able to capture and interpret these effects more accurately, given the bounded nature of prevalence rates between 0 and 1.

## 3. Results

### 3.1. Study Area and Period

Study area East consisted of 110 municipalities across 4497 km^2^. The region of Bautzen spanned 2391 km^2^, and Görlitz covered 2106 km^2^ (Supplementary Materials Figure S1). The first reported ASF case emerged on 27 October 2020 in Görlitz. The study period included 32 study months.

The first reported ASF case in study area West was detected on 5 October 2021, thus the study period comprised 20 study months. Including Dresden with a size of 328 km^2^, this study area had a size of 1781 km^2^ and consisted of 23 municipalities.

### 3.2. Data

For area East, 21,964 and for area West 2727 data records were available for analyses. For the majority of samples, no information was provided for the age or sex of the sampled animal (Table 1).

### 3.3. Data Analyses

**Figure 1:**
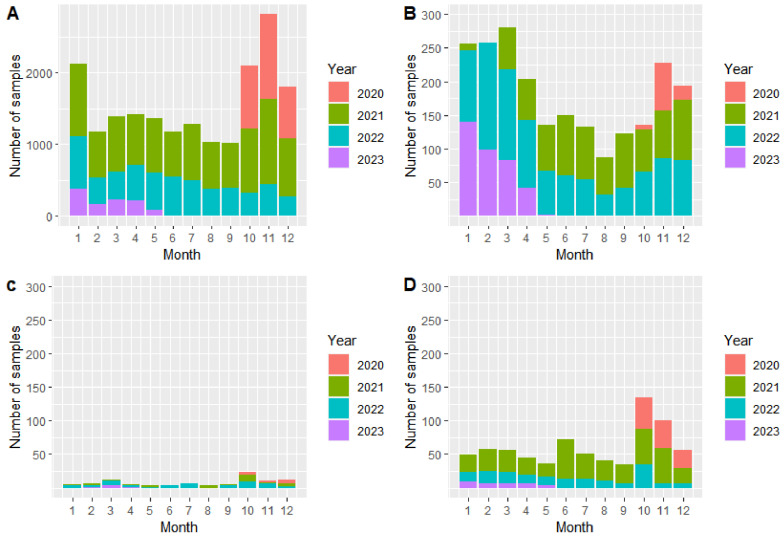
Number of investigated samples from hunted wild boar (**A**), wild boar found dead (**B**), wild boar shot sick (**C**), and wild boar that died in an RTA (**D**) in area East and per month in the years 2020–2023 (study period: 1 October 2020–17 May 2023). Please note the different scale on the *y*-axis in Figure (**A**).

**Figure 2:**
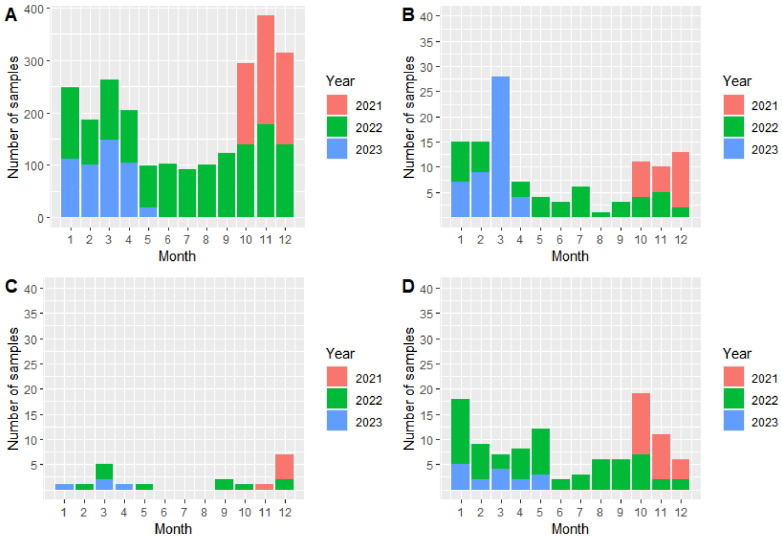
Number of investigated samples from hunted wild boar (**A**), wild boar found dead (**B**), wild boar shot sick (**C**), and wild boar that died in an RTA (**D**) in area West and per month in the years 2021–2023 (study period: 1 October 2021–17 May 2023). Please note the different scale on the *y*-axis in Figure (**A**).

In both areas, most samples originated from hunted, apparently healthy wild boar (Table 1, Figure 1A and Figure 2A). Most samples were taken in the year following the onset of the epidemic (2021 in area East and 2022 in area West) (Figure 1 and Figure 2, respectively). In area East, the number of samples from hunted wild boar declined over the years (Figure 1A), whereas in area West, in 2023, the number of samples in the individual months was similar to the numbers in 2022 (Figure 2A). The largest number of samples were taken in the winter months (Figure 1 and Figure 2).

In area East, in contrast to samples from hunted wild boar, the number of samples from wild boar found dead was higher in 2022 (Figure 1B). Particularly in the winter months, more wild boar carcasses were detected (Figure 1B). This seasonal pattern also applied for area West (Figure 2B).

A very low number of samples originated from wild boar that were shot sick (Figure 1C and Figure 2C). Particularly in area West, in total, only 20 samples came from such animals (Figure 2C). In area East, 98 samples originated from wild boar shot sick, most of them from the year 2022 (Figure 1C).

The number of samples coming from wild boar that died in an RTA was clearly higher than the number of samples from wild boar shot sick. Most samples were taken in the first months after the emergence of the disease and again in the winter months (Figure 1D and Figure 2D).

**Figure 3:**
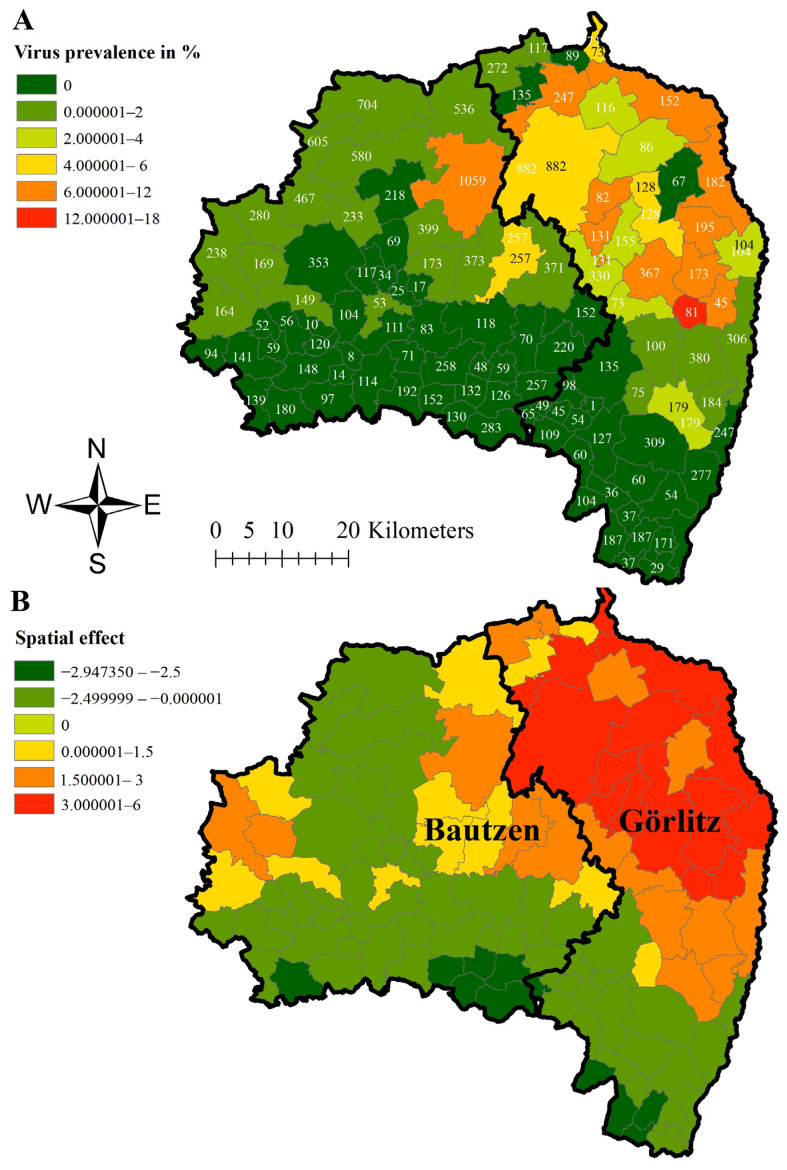
Median virus prevalence estimates of hunted wild boar in the different municipalities of study area East (districts Görlitz and Bautzen) (**A**) and median-structured spatial effect on the logit virus prevalence in hunted wild boar per municipality in study area East (districts Görlitz and Bautzen) (**B**) for the study period of 32 months. Numbers in Figure (**A**) represent the total number of samples taken in the municipalities within the study period.

Subsequently, prevalence estimates were calculated and depicted as described in Figure 3. In area East, median virus prevalence obtained from hunted wild boar was the highest (17.28%; 95% CI 9.78–27.30%) in Königshain, a small municipality in Görlitz (Figure 3A). Also, most of the other municipalities with prevalence estimates higher than 6% were located close to the border of Poland in Görlitz (Figure 3A). However, in Lohsa, a municipality in Bautzen but directly bordering Görlitz, the estimated virus prevalence in hunted wild boar was 8.12% (95% CI 6.55–9.93%) (Figure 3A).

**Figure 4:**
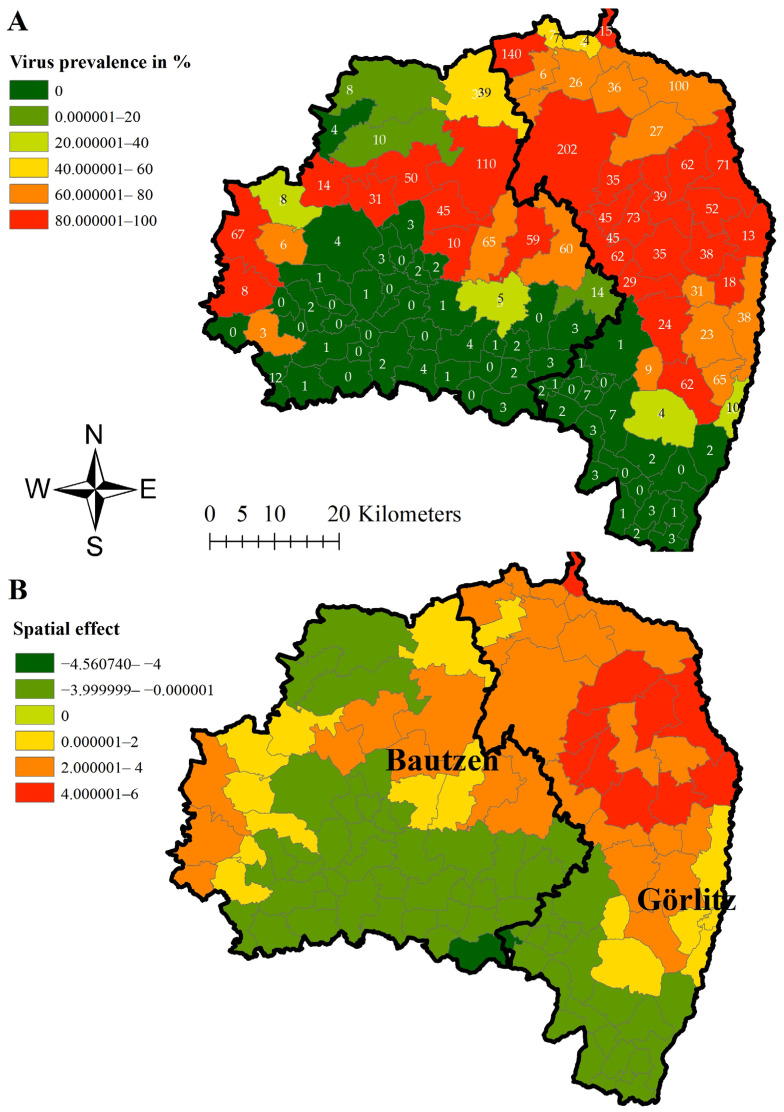
Median virus prevalence estimates of wild boar found dead in the different municipalities of in study area East (districts Görlitz and Bautzen) (**A**) and median-structured spatial effect on the logit virus prevalence in wild boar found dead per municipality in study area East (districts Görlitz and Bautzen) (**B**) for the study period of 32 months. Numbers in Figure (**A**) represent the total number of samples taken in the municipalities within the study period.

The estimated median ASFV prevalence in wild boar found dead showed values above 80% in 27 of the 110 municipalities in area East (Figure 4A). The highest prevalence (98%; 95% CI 89.35–100%) was found in Wittichenau in north-central Bautzen. Also, the two municipalities of Königsbrück (85.07; 95% CI 74.26–92.60%) and Laussnitz (87.50%; 95% CI 47.35–99.68%) in the West of Bautzen showed high virus prevalence estimates (Figure 4A).

**Figure 5:**
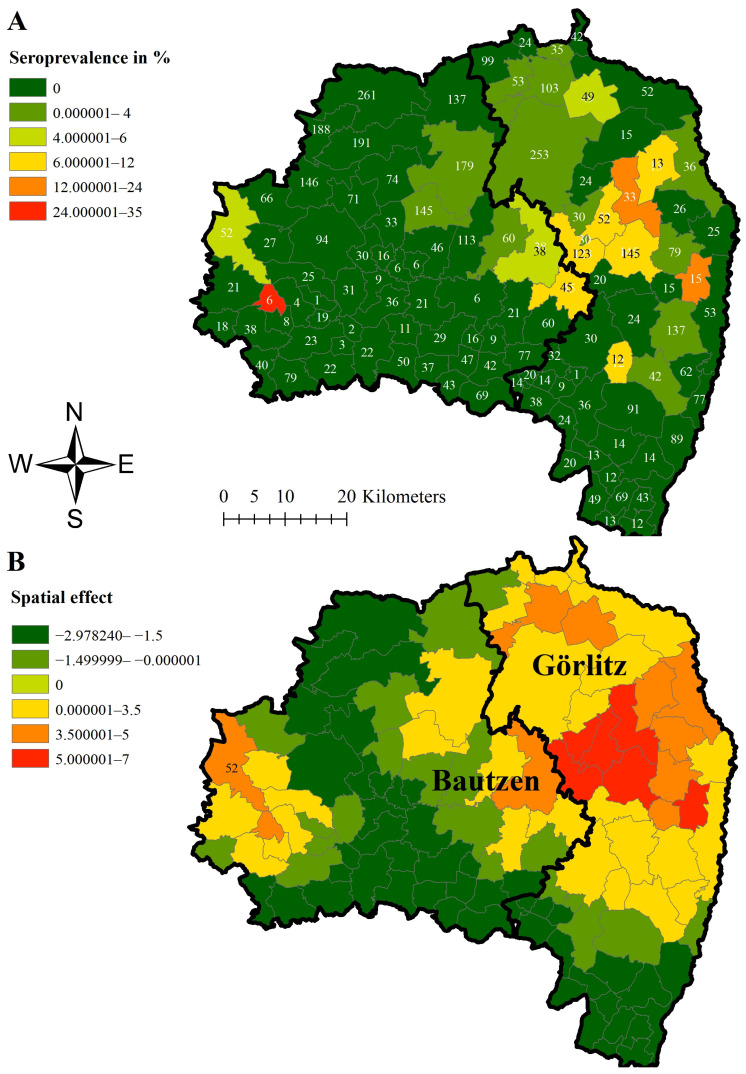
Median seroprevalence estimates of hunted wild boar in the different municipalities of study area East (districts Görlitz and Bautzen) (**A**) and median-structured spatial effect on the logit seroprevalence in hunted wild boar per municipality (**B**) for the study period of 32 months. Numbers in Figure (**A**) represent the total number of samples taken in the municipalities within the study period.

The highest seroprevalence estimate for hunted wild boar was found in Grossnaundorf (33.33%; 95% CI 4.33–77.72%), located in the West of Bautzen. However, during the study period, only six samples were serologically investigated in this municipality (Figure 5B). In 99 of the 110 municipalities, the seroprevalence estimates were lower than 5%, with partly high sample sizes, thus very narrow confidence intervals.

For all three prevalence estimations, the strongest dynamic of infection, shown by a structured spatial effect (Figure 3A, Figure 4A and Figure 5A), became evident in municipalities in Görlitz, some of them bordering Poland. Negative spatial effects were mainly seen in the municipalities centrally located in Bautzen (Figure 3B, Figure 4B and Figure 5B).

**Figure 6:**
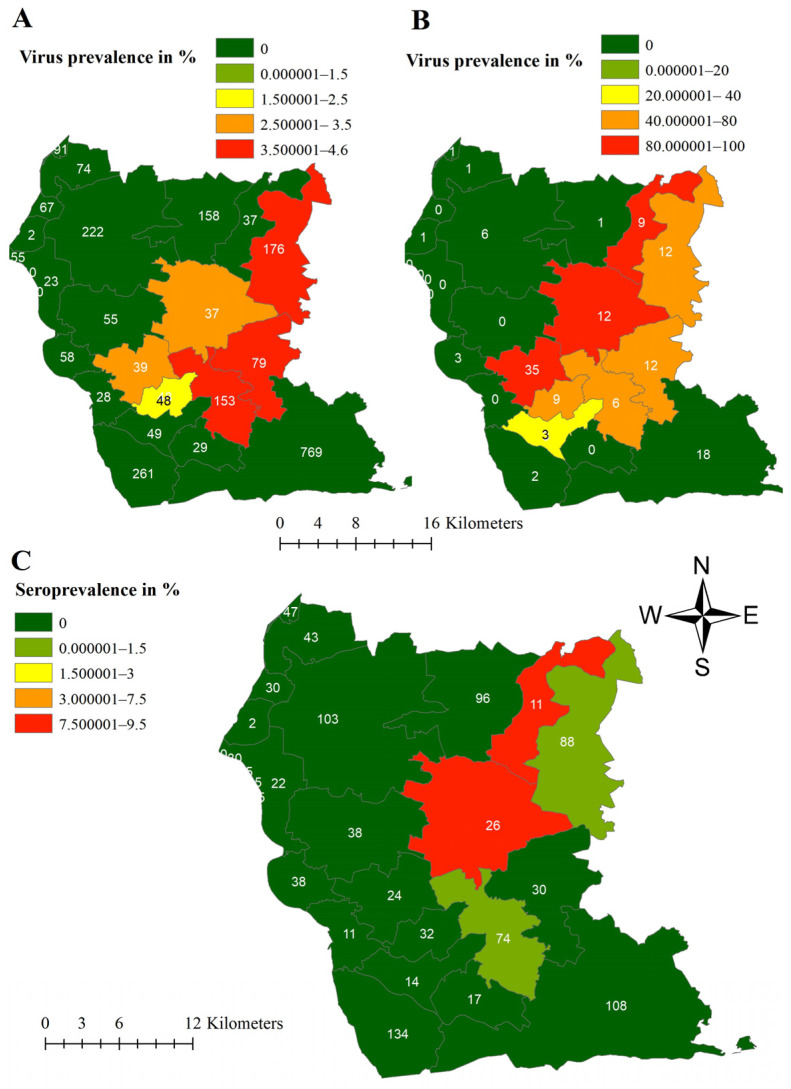
Median ASFV prevalence estimates of wild boar hunted (**A**) and found dead (**B**), and seroprevalence estimates of hunted wild boar (**C**) in the different municipalities of the district Meissen (study area West) for the study period of 20 months. Numbers on the maps represent the total number of samples taken in the municipalities within the study period.

In area West, the ASFV prevalence estimates for hunted wild boar were highest (4.55%; 95% CI 1.99–8.76%) in the municipality of Thiendorf, which is located north-east in the study area (Figure 6A). The ASFV prevalence estimates for wild boar found dead (100%; 95% CI 74.54–100%) (Figure 6B) and seroprevalence estimates for hunted wild boar (9.09%; 95% CI 0.23–41.28%) (Figure 6C) showed the highest values in Ebersbach and in Schönfeld, south and West of Thiendorf, respectively. Although several samples were investigated in the western municipalities, in most of them, no positive wild boar were detected (Figure 6). Seropositive wild boars were only found in four municipalities (Thiendorf, Ebersbach, Schönfeld, and Moritzburg) (Figure 6C).

**Figure 7:**
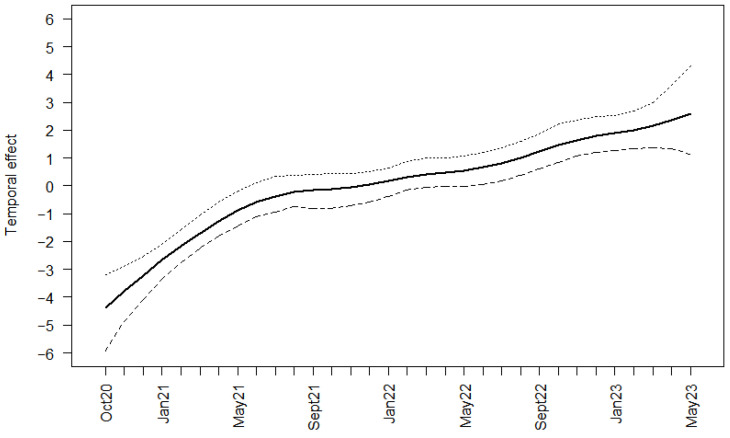
Median temporal effect on the logit prevalence for all samples of wild boar carcasses that tested ASFV-positive in area East; 95% Bayesian credible intervals (BCIs) are indicated.

In area East, the virus prevalence of hunted wild boar increased slightly with several peaks over time. The highest peak with a prevalence of 11.72% (95% CI 9.18–14.67%) emerged in June 2022. Subsequently, the prevalence decreased again, returning to levels similar to those observed at the beginning of the study period (Supplementary Materials Table S1). Adjusted for seasonal effects (Supplementary Materials Figure S2), the temporal trend of the logit prevalence of virus-positive apparently healthy, hunted wild boar showed a similar pattern. Highest values were reached in May and June 2022, also followed by a decreasing trend (Supplementary Materials Figure S3). The increase in ASFV prevalence in wild boar found dead was steeper than that for hunted wild boar, presenting a peak (92.21%; 95% CI 83.81–97.09%) in July 2021. In the following months, the prevalence estimates remained at a high level; only in August 2022, the prevalence dropped drastically (32.14%; 95% CI 15.88–52.35%), only to return to the previous level in the following months (Supplementary Materials Table S2). The temporal trend of the logit prevalence of virus-positive wild boar found dead, including the seasonal effect (Supplementary Materials Figure S4), showed a clear increase over time (Figure 7).

The seroprevalence of hunted wild boar increased only after one year of the epidemic. Several peaks, with the highest peak in July 2022 (5.23%; 95% CI 3.02–8.35%), were followed by a clear prevalence decrease (Supplementary Materials Table S3). The model analysis resulted in a slow increase in logit prevalence over time (Supplementary Materials Figure S5). The median seasonal effect is displayed in the Supplementary Materials Figure S6.

Starting to increase in the 8th study month, the prevalence estimates of wild boar found dead that were ASFV- and seropositive showed the highest value in January 2022 (66.67%; 95% CI 48.17–82.04%). In the last 12 study months, no more ASFV- and seropositive dead wild boar were detected (Supplementary Materials Table S4). The prevalence of hunted wild boar that tested positive were clearly lower than in wild boar found dead, however the highest value (5.81%; 95% CI 2.69–10.74%) had already been observed in September 2021 (Supplementary Materials Table S5).

In study area West, the estimated ASFV prevalence for hunted wild boar resulted in the highest value in May 2022 (8.89%; 95% CI 3.92–16.77%), seven months after the first case appearing in this area. In almost all subsequent months, the prevalence was zero (Supplementary Materials Table S6). In wild boar found dead, the ASFV prevalence fluctuated with several peaks. The prevalence was 100% in January 2022 (95% CI 63.06–100%) and July 2022 (95% 54.07–100%) (Supplementary Materials Table S7). The seroprevalence for hunted wild boar presented a peak in May 2022 (3.13%; 95% CI 0.08–16.22%) and in January 2023 (3.64%; 95% CI 0.44–12.53%). However, in 15 of the 20 study months, the seroprevalence was either not investigated or it was 0% (Supplementary Materials Table S8). The prevalence estimates for wild boar (hunted or found dead) that tested ASFV- and seropositive were negligible in area West (Supplementary Materials Tables S9 and S10).

## 4. Discussion

The aim of the present study was to investigate the course of ASF in the affected wild boar population in the German federal state of Saxony focusing on the different laboratory results in wild boar samples. Two different study areas were defined and analyzed separately, however both had a similar population density, which facilitates comparability [9]. The distance of more than 60 km between the epidemic in the East and the ASF cases emerging in Meissen in the West suggest a human-mediated disease introduction and no direct disease spread through infectious wild boar [9]. In the literature, the approximate distance of ASF spread is 1 to 1.5 km per month [4,22,23]. Even assuming a faster spread, the disease would not have reached such a western point within 12 months, suggesting two independent epidemiological scenarios. This hypothesis is supported by the extensive surveillance data collected between the first case in the East and the first case in Meissen, approximately one year later (Supplementary Materials, Figure S7). Despite the large number of samples analyzed, which provides a reasonable degree of confidence, no positive wild boar were detected between the two areas during this period.

All prevalence estimates are lower in study area West, which suggests that the viral load in the area is not as high as in the East. The epidemiological situation in eastern Saxony is comparable to the one in the Baltic countries or in the federal state Brandenburg [5]. In Lithuania, where disease introduction was also very likely due to the migration of infected wild boar [24], similar ASFV prevalence estimates were observed in hunted wild boar and carcasses [25]. In contrast, in Sweden, where the disease has been introduced by human activity [1,26], the ASFV prevalence of hunted wild boar in the infected zone was 0%, although within 10 months, 104 hunted animals were examined. ASFV prevalence of wild boar found dead was 54.12% [27], thus lower than in areas that face high infection pressure due to the epidemiological situation in the neighborhood, but comparable to the prevalence in Meissen. In the Czech Republic, which also experienced a point introduction in 2017, the virus prevalence of hunted and found-dead wild boar was similar to that observed in Meissen. [7]. In the meantime, the Czech Republic has experienced both epidemiological scenarios. In 2022, the disease was reintroduced in the north, likely through the migration of infected wild boar from Poland. In this scenario, the virus prevalence of hunted wild boar is more similar to the situation in study area East [28]. These findings highlight the differences between the two epidemiological scenarios, underscoring the need for tailored control measures, the associated challenges, and their long-term effectiveness.

The differences mainly arise from the longer time frame expected due to the continuous migration of infected wild boar from neighboring countries. For example, resource allocation must be carried out more cautiously, and zones should be defined more broadly.

The decision to only include data from areas that were located in restriction zone II guaranteed the best possible data basis, as the sampling and investigation of all wild boar hunted and found dead are mandatory in these areas. With regard to the descriptive analyses, it was hardly surprising that most samples originated from hunted wild boars. However, also the number of samples of wild boar involved in RTA were higher than in several other countries [27,29,30]. This could be due to an underreporting bias as, for example, in Poland, also a high number of samples originated from RTA victims [29]. In the majority of samples, no information was provided regarding the age or sex of the animal, making further analyses of these parameters difficult. Currently, there is no indication that age and sex are strongly associated with disease susceptibility. However, precise information regarding the origin of samples allows comprehensive and detailed analyses and thus a differentiated view of the epidemiology of the disease [31]. The prospect of successfully controlling the disease through a better understanding of epidemiological patterns should help motivate hunters to provide accurate information about the animal when sending samples. The seasonal sampling patterns in both areas are largely similar to those in the Baltic countries and Poland [12,32], supporting the assumption that the number of samples correlates with the hunting season. Interestingly, the number of samples from wild boar that were shot sick was very low in both study areas. These findings could be explained by fast disease progression and lethality in ASF. It can be assumed that most diseased animals will go into hiding and die before a hunter even detects them.

For the prevalence estimations, the results from samples originating from hunted wild boar and those that were killed in an RTA were combined. In the same way, samples from wild boar found dead and shot sick were analyzed together. This was performed under the assumption that hunted wild boar and RTA victims have the same probability of being infected with ASFV, as do the other two groups [30]. Seroprevalence estimates were only calculated for hunted wild boar to ensure consistent sample quality and thus reliable results. However, to evaluate the proportion of wild boar that survived the infection for at least 7–10 days [10], the prevalence estimates were also calculated for wild boar (hunted and found dead) that tested ASFV-positive and simultaneously seropositive.

The generally higher ASFV prevalence estimates for wild boar carcasses compared to the ones for hunted animals correspond to the results of previous prevalence studies [12,13,24,32] and can also be explained by the high lethality and the associated higher probability to detect the virus in wild boar carcasses.

Although the number of sampled hunted wild boar was similar in all municipalities, higher prevalence estimates were mainly observed in the eastern municipalities in the corresponding study areas. To account for spatial dependencies between neighboring municipalities and thus avoiding a false interpretation of spatial disease distribution, a hierarchical Bayesian space–time model, at least for study area East, was applied. The positive spatial effect on the logit ASFV prevalence in the four western municipalities in area East (Figure 3) indicates that the disease tends to move towards these municipalities. This interpretation is supported by the high ASFV prevalence estimates for wild boar found dead in these four municipalities. The spatial effect on the logit ASFV prevalence of wild boar found dead is not surprising and coincides with the ASFV prevalence estimations of wild boar found dead. The high seroprevalence in Grossnaundorf has to be interpreted considering the low sample size and thus the wide confidence interval in this municipality. However, detecting few seropositive wild boar in this area and thus animals that probably survived the infection is not surprising as also the ASFV prevalence of dead wild boar was high in these municipalities. Also, in the other municipalities, seroprevalence estimates were particularly high in the municipalities in which the ASFV prevalence was also higher.

In area West, the different prevalence patterns resembled each other. The virus first appeared in Radeburg, which is located on the eastern border of the Meissen district.

However, unlike the neighboring communities to the West and north, the seroprevalence in this area is zero, indicating that the infection was concentrated in the surrounding communities.

The temporal trends of ASFV- and seroprevalence were comparable to other affected countries. Similar studies were performed in Baltic countries, and the increase in ASFV prevalence followed by an increase in seroprevalence were comparable [1,12,13,14]. In Baltic countries, where the trend over more than 70 study months was investigated, it was observed that the temporal effect decreased after approx. three years of the epidemic. Thus, it could be assumed that, also in Saxony, the trend will decline in the near future.

The seasonal prevalence estimates in the different study months and the two study areas did not follow a consistent pattern. In Poland, prevalence rather peaked in the winter month [1,29]. However, Rogoll et al. [33] and also Stahl et al. [1] demonstrated in their studies that seasonal prevalence patterns of wild boar were not consistent in the different European countries, which fits the pattern observed in Saxony. When interpreting changes in prevalence estimates over time, it is important to always consider the sample size and the resulting confidence intervals. Seroprevalence estimates could only be calculated for a defined period of time. Saxony started with serological investigations for ASFV-specific antibodies in March 2021 and stopped two years later by the end of February 2023. The increase in seroprevalence over time is not surprising, as it is known that affected wild boar seroconvert and can survive the infection [34,35]. Thus, the increase is the result of an accumulation of surviving animals and was observed in other countries [12]. Determining the antibody status of sampled wild boar is crucial to obtain an interpretable picture of disease epidemiology. Furthermore, comparing the course of seroprevalence estimates with the one in other countries enables scientists to draw potential conclusions regarding the characteristic of the circulating virus, and thus to potentially adapt, change, or emphasize recommendations regarding disease control measures. However, as discussed by Schulz et al. [36], currently, the case definition of the WOAH includes seropositive animals. This means that, even in case of missing ASFV-positive animals and the presence of seropositive samples, an affected country cannot be declared free from the disease. Accordingly, it is, to a certain extent, the logical consequence that countries are not particularly keen to continue serological testing in the long term. In order to motivate countries to continue serological testing and thus to enable the gain of knowledge regarding ASF and its course in wild boar populations, it is probably necessary to discuss, politically, the options to deal with seropositive wild boar in areas that have already been affected by ASF. This is particularly true as there is now sufficient evidence that these animals play only a negligible role, if any, in the spread of the disease [35,37,38,39]

The present study illustrates yet again the importance of analyzing the disease pattern in different countries and regions. Only by constantly increasing our understanding of ASF and the attempt to close as many knowledge gaps as possible can the successful global control of ASF become a reality.

## Figures and Tables

**Table 1:** Number of wild boar samples in study area East (in total 21,964 samples) and study area West (in total 2727) divided by age, sex, origin of sample, and the individual test results. Na = no data available.

	Age (Years)				Sex			Origin of Sample					Test Results			
	Na	0–1	1–2	>2	Na	Female	Male	Na	Hunted	Found Dead	Shot Sick	RTA	Na	ASFV-Positive	Antibody-Positive (Seropositive)	ASFV- and Seropositive
East	18,733	1400	1378	453	19,220	1574	1170	200	18,703	2229	98	734	225	1795	67	197
West	2713	8	2	4	2716	6	5	56	2415	129	20	107	19	93	5	6

## Data Availability

The original data used for the analyses can be obtained from the corresponding authors after approval by the responsible institutions.

## Supplementary Materials

The following supporting information can be downloaded at: https://www.mdpi.com/article/10.3390/v16121894/s1, Table S1: Median temporal effect on the logit prevalence for all samples of wild boar carcasses that tested ASFV-positive in area East; 95% Bayesian credible intervals (BCIs) are indicated. Table S2: ASF virus prevalence of wild boar found dead and shot sick in area East and per study month, including the mean, maximum, and minimum virus prevalence values. Table S3: Seroprevalence of hunted wild boar and wild boar that died in RTA in area East and per study month, including the mean, maximum, and minimum seroprevalence. NA = no data available. Table S4: Prevalence of wild boar found dead and shot sick that tested positive for the ASFV genome and for ASF-specific antibodies in area East and per study month, including the mean, maximum, and minimum virus prevalence. NA = no data available. Table S5: Prevalence of wild boar hunted and wild boar that died in RTA that tested positive for the ASFV genome and for ASF-specific antibodies in area East and per study month, including the mean, maximum, and minimum virus prevalence. NA = no data available. Table S6: ASF virus prevalence for hunted wild boar and wild boar that died in RTA in area West and per study month, including the mean, maximum, and minimum virus prevalence. Table S7: ASF virus prevalence for wild boar found dead and shot sick in area West and per study month, including the mean, maximum, and minimum virus prevalence. NA = no data available. Table S8: Seroprevalence for hunted wild boar and wild boar that died in RTA in area West and per study month, including the mean, maximum, and minimum seroprevalence. NA = no data available. Table S9: Prevalence of wild boar hunted and wild boar that died in RTA that tested positive for the ASFV genome and for ASF-specific antibodies in area West and per study month, including the mean, maximum, and minimum virus prevalence. NA = no data available. Table S10: Prevalence of wild boar found dead and shot sick that tested positive for the ASFV genome and for ASF-specific antibodies in area West and per study month, including the mean, maximum, and minimum virus prevalence. NA = no data available. Figure S1: Map of Germany with the German federal states, the districts, and study area East (blue) and study area West (green). Figure S2. Median seasonal effect on the logit prevalence of samples obtained from hunted wild boar from area East that tested ASFV-positive; 95% Bayesian credible intervals (BCIs) are indicated. Figure S3. Median temporal effect on the logit prevalence for all samples of hunted wild boar from area East that tested ASFV-positive; 95% Bayesian credible intervals (BCIs) are indicated. Figure S4. Median seasonal effect on the logit prevalence of samples obtained from wild boar found dead in area East that tested ASFV-positive; 95% Bayesian credible intervals (BCIs) are indicated. Figure S5. Median temporal effect on the logit prevalence for all samples of hunted wild boar from area East that tested seropositive; 95% Bayesian credible intervals (BCIs) are indicated. Figure S6. Median seasonal effect on the logit prevalence of samples obtained from wild boar found dead in area East that tested ASFV-positive; 95% Bayesian credible intervals (BCIs) are indicated. Figure S7. The federal state Saxony and study area East (districts Bautzen and Görlitz) and study area West (district Meißen). Blue dots represent all ASF-positive wild boars sampled between 30.10.2020 and 13.10.2021. Green dots represent all ASF-negative wild boar samples from the same period.
