# Supplementary material for: African Swine Fever in Saxony—Disease Dynamics
Source: Viruses. 2024 Dec 9;16(12):1894. doi: 10.3390/v16121894 (PMC11680337; doi:10.3390/v16121894)
Supplement: Supplementary file 1 [file viruses-16-01894-s001.zip › viruses-3364519-supplementary.pdf]

Table S1. Median temporal effect on the logit prevalence for all samples of wild boar carcasses that tested ASFV-positive in area East, 95% Bayesian credible intervals (BCI) are indicated.

Study month	Virus prevalence in %	Lower 95% CI in %	Upper 95% CI in %	Total number of investigated samples
1	0.11	0.00	0.59	942
2	0.00	0.00	0.30	1,227
3	0.00	0.00	0.50	741
4	0.10	0.00	0.54	1,038
5	0.30	0.04	1.08	668
6	0.49	0.13	1.26	809
7	2.07	1.16	3.38	726
8	0.25	0.03	0.91	788
9	2.06	1.13	3.43	680
10	4.29	3.01	5.92	815
11	1.90	1.02	3.23	683
12	5.48	3.87	7.51	657
13	1.06	0.51	1.94	945
14	1.85	1.17	2.76	1,245
15	0.95	0.41	1.85	846
16	2.64	1.62	4.05	757
17	2.04	0.89	3.98	392
18	2.74	1.37	4.84	402
19	1.58	0.68	3.09	507
20	3.95	2.46	5.98	531
21	11.72	9.18	14.67	563
22	5.84	3.97	8.23	514
23	0.26	0.00	1.44	385
24	0.77	0.16	2.22	392
25	1.14	0.31	2.88	352
26	1.12	0.36	2.60	446
27	2.95	1.28	5.73	271
28	0.78	0.16	2.27	383
29	1.19	0.14	4.23	168
30	0.00	0.00	1.53	240
31	2.23	0.73	5.13	224
32	0.00	0.00	4.11	88

Mean prev in %	Maximum prev in %	Minimum prev in %
1.93	11.72	0

Table S2. ASF virus prevalence in wild boar found dead and shot sick in area East and per study month, including the mean, maximum and minimum virus prevalence.

Study month	Virus prevalence in %	Lower 95% CI in %	Upper 95% CI in %	Total number of investigated samples
1	0.00	0.00	28.49	11
2	29.17	16.95	44.06	48
3	9.52	1.17	30.38	21
4	58.33	27.67	84.83	12
5	74.47	59.65	86.06	47
6	65.57	52.31	77.27	61
7	64.52	51.34	76.26	62
8	65.28	53.14	76.12	72
9	81.82	72.16	89.24	88
10	92.21	83.81	97.09	77
11	72.88	59.73	83.64	59
12	77.50	66.79	86.09	80
13	82.19	71.47	90.16	73
14	78.08	66.86	86.92	73
15	77.66	67.90	85.61	94
16	81.48	72.86	88.31	108
17	86.88	80.64	91.69	160
18	82.98	75.74	88.78	141
19	71.13	61.05	79.89	97
20	60.94	47.93	72.90	64
21	86.44	75.02	93.96	59
22	80.70	68.09	89.95	57
23	32.14	15.88	52.35	28
24	77.50	61.55	89.16	40
25	78.69	66.32	88.14	61
26	92.54	83.44	97.53	67
27	93.15	84.74	97.74	73
28	93.58	87.22	97.38	109
29	89.74	80.79	95.47	78
30	65.63	52.70	77.05	64
31	90.32	74.25	97.96	31
32	100.00	2.50	100.00	1

Mean prev in %	Maximum prev in %	Minimum prev in %
71.66	100	0

Table S3. Seroprevalence in hunted wild boar and wild boar died in RTA in area East and per study month, including the mean, maximum and minimum seroprevalence. NA = no data available.

Study month	Seroprevalence in %	Lower 95% CI in %	Upper 95% CI in %	Total number of investigated samples
1	NA	NA	NA	0
2	NA	NA	NA	0
3	NA	NA	NA	0
4	NA	NA	NA	0
5	NA	NA	NA	0
6	0	0	33.63	9
7	0	0	4.99	72
8	0	0	11.94	29
9	0	0	3.69	98
10	0	0	1.86	197
11	0	0	2.46	148
12	0	0	2.35	155
13	0	0	1.89	193
14	3.70	1.21	8.43	135
15	1.29	0.52	2.64	542
16	0.00	0	0.70	522
17	0.36	0	1.97	280
18	4.76	0.99	13.29	63
19	2.41	1.05	4.69	332
20	1.52	0.50	3.52	328
21	0.91	0.19	2.63	330
22	5.23	3.02	8.35	306
23	1.10	0.23	3.18	273
24	1.06	0.22	3.07	283
25	1.71	0.47	4.32	234
26	0.31	0	1.70	326
27	0.50	0.01	2.74	201
28	1.56	0.43	3.94	257
29	1.23	0.03	6.69	81
30	NA	NA	NA	0
31	NA	NA	NA	0
32	NA	NA	NA	0

Mean prev in %	Maximum prev in %	Minimum prev in %
1.15	5.23	0

Table S4. Prevalence of wild boar found dead and shot sick that were tested positive for ASFV genome and for ASF-specific antibodies in area East and per study month, including the mean, maximum and minimum virus prevalence. NA = no data available.

Study month	Virus and seroprevalence in %	Lower 95% CI in %	Upper 95% CI in %	Total number of investigated samples
1	NA	NA	NA	0
2	NA	NA	NA	0
3	NA	NA	NA	0
4	0	0	97.50	1
5	NA	NA	NA	0
6	0	0	84.19	2
7	0	0	84.19	2
8	0	0	97.50	1
9	13.33	1.66	40.46	15
10	16.67	4.74	37.38	24
11	38.71	21.85	57.81	31
12	38.24	22.17	56.44	34
13	56.00	34.93	75.60	25
14	60.00	32.29	83.66	15
15	55.00	38.49	70.74	40
16	66.67	48.17	82.04	33
17	48.78	32.88	64.87	41
18	47.37	30.98	64.18	38
19	NA	NA	NA	0
20	100	2.50	100.00	1
21	0	0	97.50	1
22	NA	NA	NA	0
23	NA	NA	NA	0
24	NA	NA	NA	0
25	0	0	84.19	2
26	0	0	97.50	1
27	NA	NA	NA	0
28	0	0	84.19	2
29	NA	NA	NA	0
30	NA	NA	NA	0
31	NA	NA	NA	0
32	NA	NA	NA	0

Mean prev in %	Maximum prev in %	Minimum prev in %
28.46	100	0

Table S5. Prevalence of wild boar hunted and wild boar died in RTA that were tested positive for ASFV genome and for ASF-specific antibodies in area East and per study month, including the mean, maximum and minimum virus prevalence. NA = no data available.

Study month	Virus and seroprevalence in %	Lower 95% CI in %	Upper 95% CI in %	Total number of investigated samples
1	NA	NA	NA	0
2	NA	NA	NA	0
3	NA	NA	NA	0
4	NA	NA	NA	0
5	NA	NA	NA	0
6	0.00	0.00	33.63	9
7	0.00	0.00	4.99	72
8	0.00	0.00	11.94	29
9	2.04	0.25	7.18	98
10	3.05	1.13	6.51	197
11	4.73	1.92	9.50	148
12	5.81	2.69	10.74	155
13	1.55	0.32	4.48	193
14	3.70	1.21	8.43	135
15	0.55	0.11	1.61	542
16	1.53	0.66	3.00	522
17	0.36	0.00	1.97	280
18	1.59	0.04	8.53	63
19	0.90	0.19	2.62	332
20	1.22	0.33	3.09	328
21	0.00	0.00	1.11	330
22	0.65	0.08	2.34	306
23	0.00	0.00	1.34	273
24	0.00	0.00	1.30	283
25	0.85	0.10	3.05	234
26	0.61	0.07	2.20	326
27	1.00	0.12	3.55	201
28	0.00	0.00	1.43	257
29	0.00	0.00	4.45	81
30	NA	NA	NA	0
31	NA	NA	NA	0
32	NA	NA	NA	0

Mean prev in %	Maximum prev in %	Minimum prev in %
1.26	5.81	0

Table S6. ASF virus prevalence in hunted wild boar and wild boar died in RTA in area West and per study month, including the mean, maximum and minimum virus prevalence.

Study month	Virus prevalence in %	Lower 95% CI in %	Upper 95% CI in %	Total number of investigated samples
1	0.60	0.02	3.29	167
2	0.92	0.11	3.29	217
3	1.67	0.35	4.79	180
4	0.66	0.02	3.63	151
5	0.00	0.00	3.89	93
6	0.85	0.02	4.67	117
7	0.00	0.00	3.42	106
8	8.89	3.92	16.77	90
9	2.88	0.60	8.20	104
10	0.00	0.00	3.81	95
11	0.00	0.00	3.39	107
12	0.00	0.00	2.82	129
13	0.00	0.00	2.49	146
14	0.00	0.00	2.04	179
15	0.00	0.00	2.58	141
16	0.00	0.00	3.16	115
17	0.98	0.02	5.34	102
18	0.00	0.00	2.38	153
19	0.00	0.00	3.39	107
20	0.00	0.00	16.11	21

Mean prev in %	Maximum prev in %	Minimum prev in %
0.87	8.89	0

Table S7. ASF virus prevalence in wild boar found dead and shot sick in area West and per study month, including the mean, maximum and minimum virus prevalence. NA = no data available.

Study month	Virus prevalence in %	Lower 95% CI in %	Upper 95% CI in %	Total number of investigated samples
1	66.67	22.28	95.67	6
2	66.67	22.28	95.67	6
3	73.33	44.90	92.21	15
4	100.00	63.06	100.00	8
5	66.67	22.28	95.67	6
6	26.67	7.79	55.10	15
7	33.33	0.84	90.57	3
8	60.00	14.66	94.73	5
9	33.33	0.84	90.57	3
10	100.00	54.07	100.00	6
11	0.00	0.00	97.50	1
12	0.00	0.00	60.24	4
13	60.00	14.66	94.73	5
14	0.00	0.00	70.76	3
15	0.00	0.00	60.24	4
16	33.33	4.33	77.72	6
17	42.86	9.90	81.59	7
18	92.31	74.87	99.05	26
19	0.00	0.00	84.19	2
20	NA	NA	NA	0

Mean prev in %	Maximum prev in %	Minimum prev in %
45.01	100	0

Table S8. Seroprevalence in hunted wild boar and wild boar died in RTA in area West and per study month, including the mean, maximum and minimum seroprevalence. NA = no data available.

Study month	Seroprevalence in %	Lower 95% CI in %	Upper 95% CI in %	Total number of investigated samples
1	NA	NA	NA	0
2	0.00	0.00	70.76	3
3	0.00	0.00	2.58	141
4	0.91	0.02	4.96	110
5	0.00	0.00	5.36	67
6	0.00	0.00	70.76	3
7	0.00	0.00	6.72	53
8	3.13	0.08	16.22	32
9	0.00	0.00	6.16	58
10	1.67	0.04	8.94	60
11	0.00	0.00	5.69	63
12	0.00	0.00	6.27	57
13	0.00	0.00	4.11	88
14	0.00	0.00	3.39	107
15	0.00	0.00	4.25	85
16	3.64	0.44	12.53	55
17	0.00	0.00	8.60	41
18	NA	NA	NA	0
19	NA	NA	NA	0
20	NA	NA	NA	0

Mean prev in %	Maximum prev in %	Minimum prev in %
0.58	3.64	0

Table S9. Prevalence of wild boar hunted and wild boar died in RTA that were tested positive for ASFV genome and for ASF-specific antibodies in area West and per study month, including the mean, maximum and minimum virus prevalence. NA = no data available.

Study month	Virus and seroprevalence in %	Lower 95% CI in %	Upper 95% CI in %	Total number of investigated samples
1	NA	NA	NA	0
2	0.00	0.00	70.76	3
3	1.42	0.17	5.03	141
4	0.91	0.02	4.96	110
5	0.00	0.00	5.36	67
6	0.00	0.00	70.76	3
7	0.00	0.00	6.72	53
8	0.00	0.00	10.89	32
9	1.72	0.04	9.24	58
10	0.00	0.00	5.96	60
11	0.00	0.00	5.69	63
12	0.00	0.00	6.27	57
13	0.00	0.00	4.11	88
14	0.00	0.00	3.39	107
15	0.00	0.00	4.25	85
16	0.00	0.00	6.49	55
17	0.00	0.00	8.60	41
18	NA	NA	NA	0
19	NA	NA	NA	0
20	NA	NA	NA	0

Mean prev in %	Maximum prev in %	Minimum prev in %
0.25	1.72	0

Table S10. Prevalence of wild boar found dead and shot sick that were tested positive for ASFV genome and for ASF-specific antibodies in area West and per study month, including the mean, maximum and minimum virus prevalence. NA = no data available.

Study month	Virus and seroprevalence in %	Lower 95% CI in %	Upper 95% CI in %	Total number of investigated samples
1	NA	NA	NA	0
2	NA	NA	NA	0
3	50.00	6.76	93.24	4
4	NA	NA	NA	0
5	0.00	0	97.50	1
6	0.00	0	84.19	2
7	NA	NA	NA	0
8	NA	NA	NA	0
9	NA	NA	NA	0
10	NA	NA	NA	0
11	NA	NA	NA	0
12	NA	NA	NA	0
13	0.00	0	84.19	2
14	NA	NA	NA	0
15	NA	NA	NA	0
16	NA	NA	NA	0
17	NA	NA	NA	0
18	NA	NA	NA	0
19	NA	NA	NA	0
20	NA	NA	NA	0
**Mean prev in %**	**Maximum prev in %**	**Minimum prev in %**		
2.5	50	0		

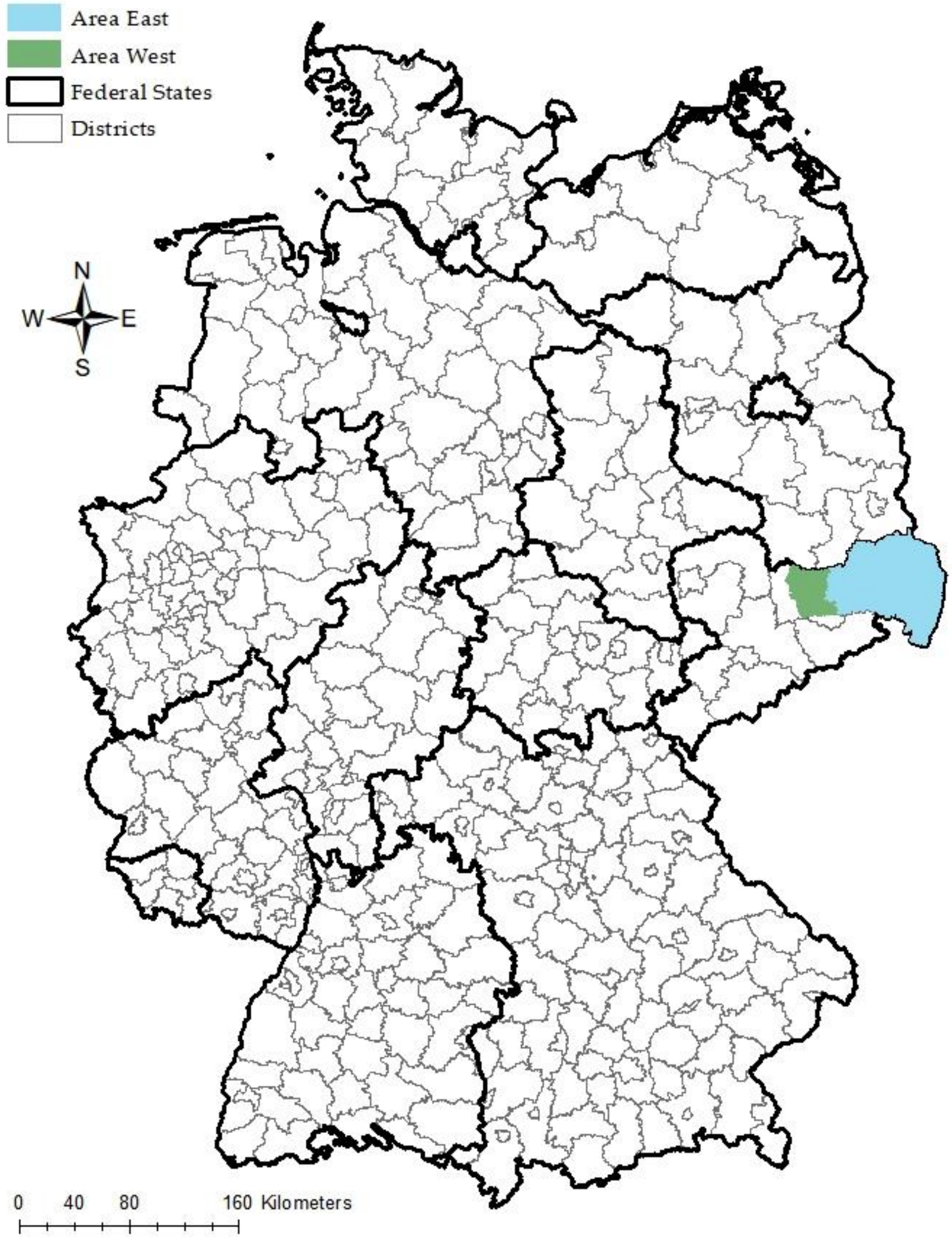

Figure S1. Map of Germany with the German federal states, the districts and study area East (blue) and study area West (green).

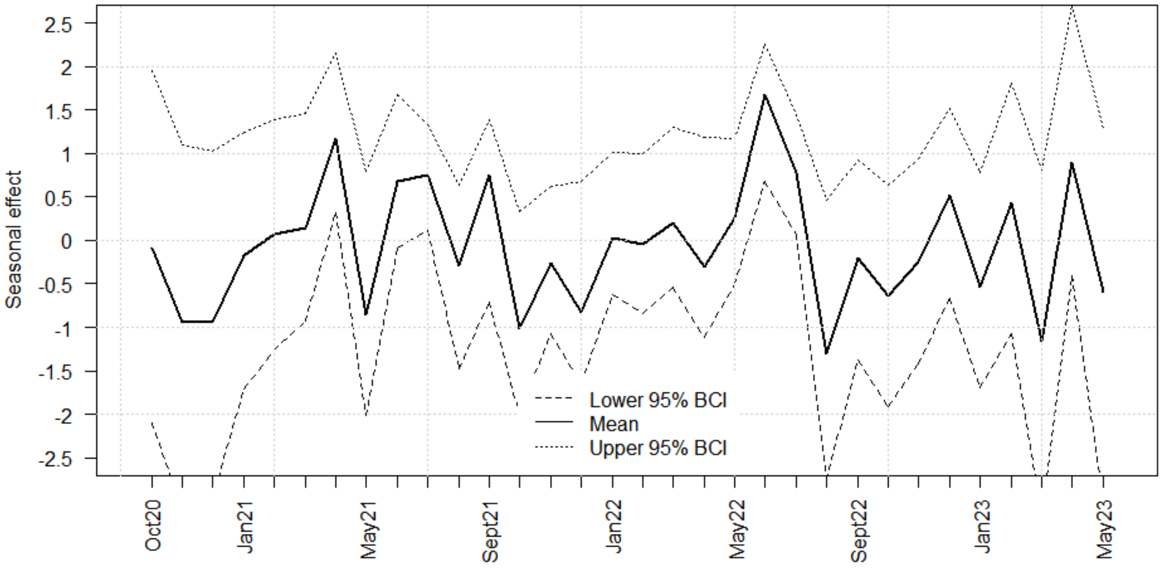

Figure S2. Median seasonal effect on the logit prevalence of samples obtained from hunted wild boar from area East that tested ASFV-positive. 95% Bayesian credible intervals (BCI) are indicated.

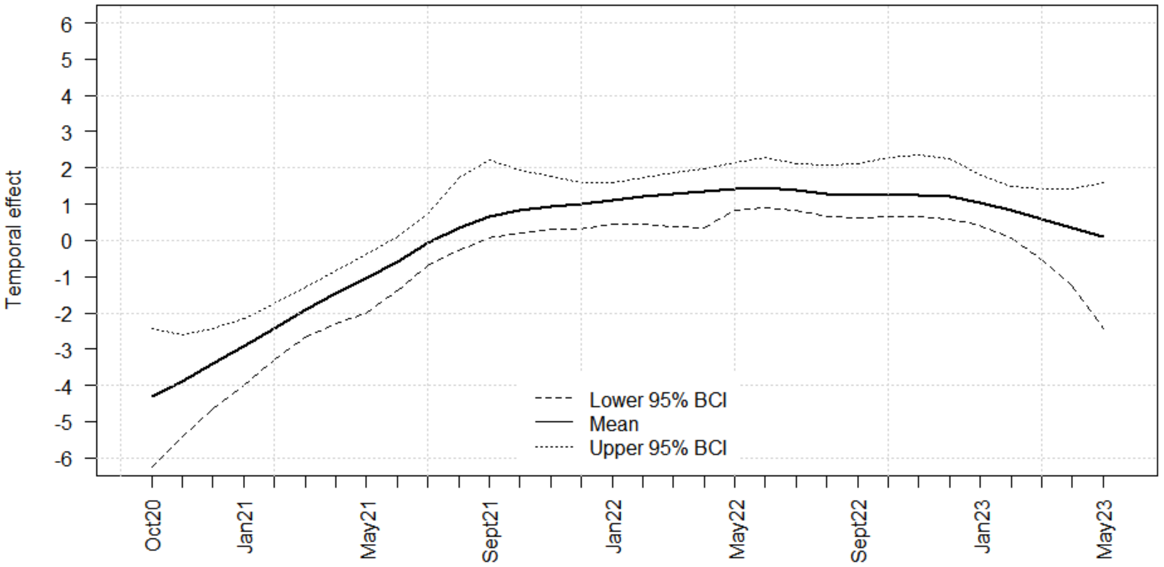

Figure S3. Median temporal effect on the logit prevalence for all samples of hunted wild boar from area East that tested ASFV-positive, 95% Bayesian credible intervals (BCI) are indicated.

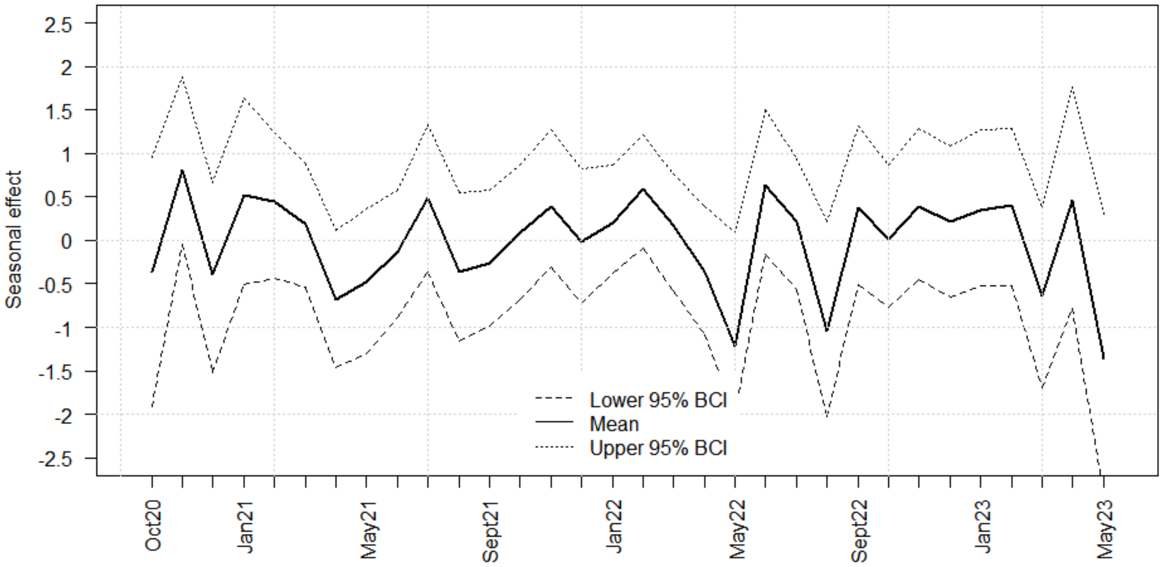

Figure S4. Median seasonal effect on the logit prevalence of samples obtained from wild boar found dead from area East that tested ASFV-positive. 95% Bayesian credible intervals (BCI) are indicated.

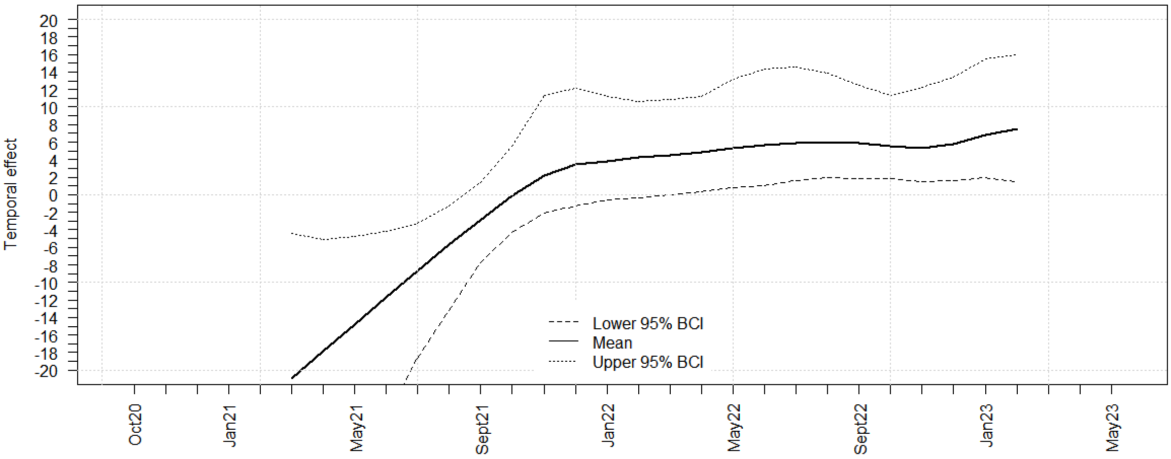

Figure S5. Median temporal effect on the logit prevalence for all samples of hunted wild boar from area East that tested seropositive, 95% Bayesian credible intervals (BCI) are indicated.

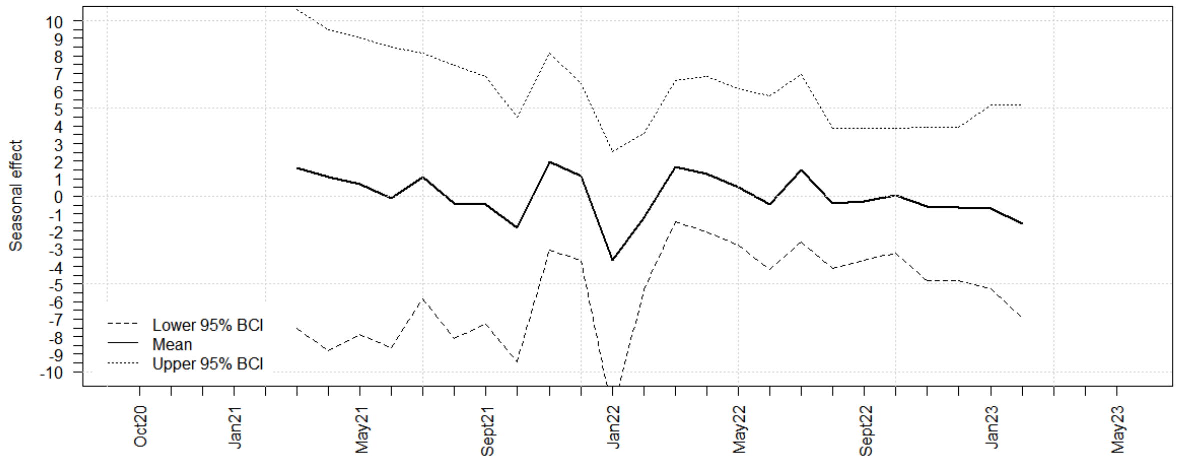

Figure S6. Median seasonal effect on the logit prevalence of samples obtained from wild boar found dead from area East that tested ASFV-positive. 95% Bayesian credible intervals (BCI) are indicated.

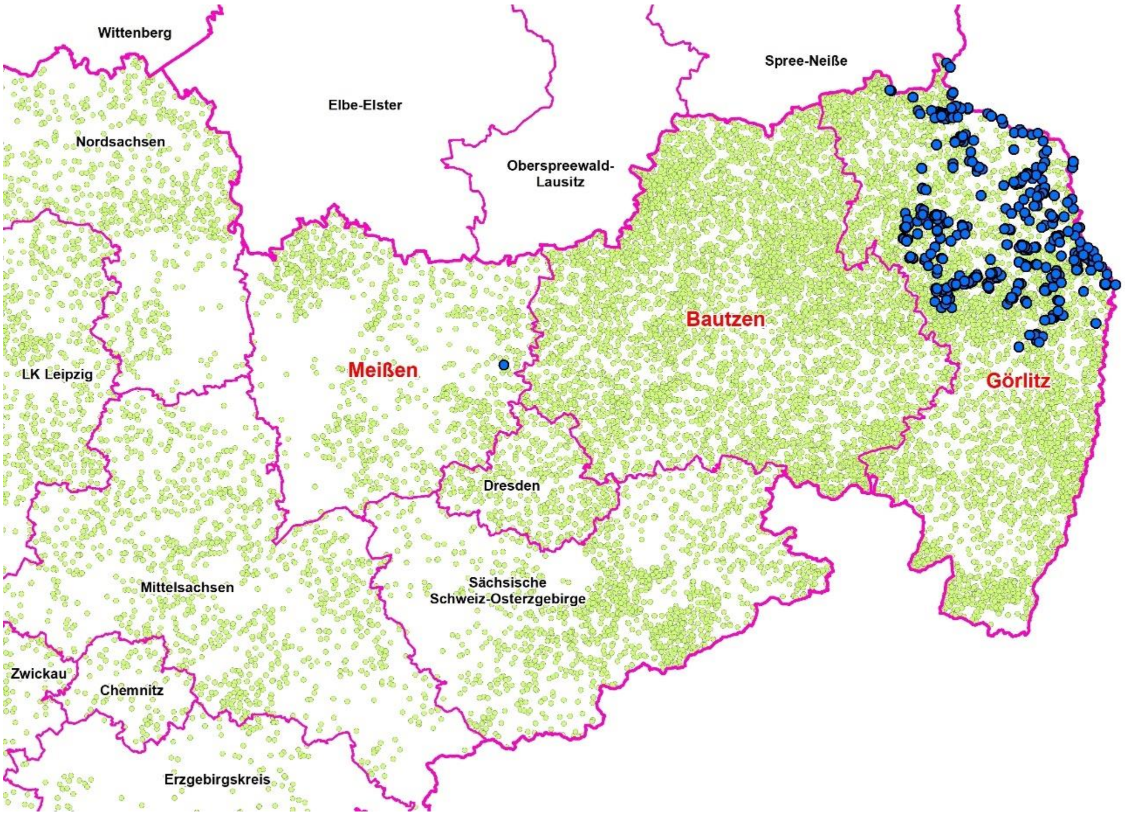

Figure S7. The federal state Saxony and study area East (districts Bautzen and Görlitz) and study area West (district Meissen). Blue dots represent all ASF-positive wild boar between 30.10.2020 and 13.10.2021. Green dots represent all ASF-negative wild boar samples in the same period.
